# Diabetes Mellitus Type 2 Patients with Abdominal Obesity Are Prone to Osteodysfunction: A Cross-Sectional Study

**DOI:** 10.1155/2023/3872126

**Published:** 2023-04-17

**Authors:** Hui Guo, Zengmei An, Ningjian Wang, Shaohong Ge, Jian Cai, Shiyan Yu, Ying Zhou, Rong Ying, Kexi Zha, Tao Gu, Yan Zhao, Yingli Lu

**Affiliations:** ^1^Institute and Department of Endocrinology and Metabolism, Huangpu Branch of Shanghai Ninth People's Hospital, Shanghai, China; ^2^Institute and Department of Endocrinology and Metabolism, Shanghai Ninth People's Hospital, Shanghai Jiao Tong University School of Medicine, Shanghai, China

## Abstract

**Introduction:**

The interaction between diabetes, obesity, and bone metabolism was drawing increasing public attention. However, the osteometabolic changes in diabetes mellitus type 2 (T2DM) patients with abdominal obesity have not been fully revealed. This study is aimed at investigating the association between abdominal obesity indices and bone turnover markers among T2DM participants.

**Methods:**

4351 subjects were involved in the METAL study. Abdominal obesity indices included neck, waist, and hip circumference, visceral adiposity index (VAI), lipid accumulation product (LAP), waist-to-hip ratio (WHR), and Chinese visceral adiposity index (CVAI). They were applied to elucidate the nexus between *β*-C-terminal telopeptide (*β*-CTX), osteocalcin (OC), and intact N-terminal propeptide of type I collagen (P1NP).

**Results:**

Abdominal obesity indices were strongly negatively associated with *β*-CTX and OC. Among males, five indices were negatively correlated with *β*-CTX (BMI, WC, LAP, WHR, and CVAI) and OC (BMI, NC, WC, WHR, and CVAI). There were no significant associations with P1NP. Among females, all eight indices were negatively associated with *β*-CTX. Seven indices were negatively related to OC (BMI, NC, WC, HC, LAP, WHR, and CVAI). The VAI was negatively correlated with P1NP.

**Conclusions:**

The present study demonstrated that in T2DM, abdominal obesity had an obviously negative correlation with bone metabolism. Abdominal obesity indices were significantly negatively associated with skeletal destruction (*β*-CTX) and formation (OC). In routine clinical practice, these easily obtained indices could be used as a preliminary screening method and relevant factors for osteodysfunction incidence risk at no additional cost and may be of particular value for postmenopausal women in T2DM populations.

## 1. Introduction

Diabetes mellitus type 2 (T2DM), obesity, and osteoporosis were reaching epidemic proportions worldwide. They have been identified as public health issues with increased mortality due to their high prevalence and severe metabolic complications [1].

The effect of obesity on osteoporosis remained controversial [2, 3]. The majority of studies have suggested that obesity had a protective effect against excessive bone loss with aging [4, 5] which may be due to material stimulation during bone formation [6, 7] and the physical protection provided by the adipose tissue, thus preventing accidental fall-induced fractures [4, 8]. In contrast to the studies mentioned above, others have reported a negative impact of obesity on skeletal health [9–11]. Physical inactivity, nutritional imbalance [12, 13], and genetic disorders were common risk factors for the pathological physiology of obesity and osteoporosis [14]. In a cross-sectional study involving 1434 women older than 45 years, for instance, a significantly negative association between bone mineral density (BMD) and waist circumference (WC) was found [11].

However, pathogenic fat depots, notably in the abdomen, have been hypothesized to contribute more to bone disability [15–17]. A recent study on more than 650000 adults showed that a rise in WC led to an obvious and rapid increase in fracture risk, independent of the body mass index (BMI) level (three groups: normal, overweight, and obese) [18]. Another multicenter, observational study called the “Osteoarthritis Initiative,” which included 2210 participants aged 67.1-69 years, found a relationship between visceral obesity and skeletal joint functional incapacity in people with normal BMI [19, 20].

However, the interaction between T2DM and bone metabolism was complex, and it is unclear whether hyperglycemia increased or decreased BMD [3]. One study described the differences in the blood glucose levels among osteoporosis, low BMD, and normal BMD [21]. Furthermore, an investigation in 30252 nondiabetic women aged ≥40 years showed that an index of abdominal fat measured by spine dual-energy X-ray absorptiometry (DXA) scans performed for osteoporosis risk assessment could predict diabetes risk [22]. However, a meta-analysis results also showed that abnormal glucose regulation was not significantly correlated with bone metabolism [23].

Due to the hysteresis of BMD detection, the development and course of osteodysfunction over time cannot be predicted. Currently, circulating bone turnover markers (BTMs) were widely detected to evaluate changes in bone formation and resorption, estimate the therapeutic effect on osteoporosis, and forecast the processes of bone metabolic abnormalities [24–26]. International consensus guidelines recommend the assessment of three BTMs: *β*-C-terminal cross-linking telopeptide of type I collagen (*β*-CTX) (released by osteoclasts during bone resorption), osteocalcin (OC), and procollagen type 1 N-terminal propeptide (P1NP) (produced by osteoblasts during bone formation) [27, 28].

The aim of this study was to investigate the relationship between abdominal obesity and the three BTMs among Chinese people with T2DM based on a large community-sourced sample.

## 2. Methods

### 2.1. Study Design and Participants

The subjects enrolled in our research were a subset from a population-based study, named the METAL study (Environmental Pollutant Exposure and Metabolic Diseases in Shanghai) [29, 30]. The program is aimed at revealing the complications of metabolic diseases and relevant factors among seven communities in Shanghai, China, from May to August 2018 (Trial registration ChiCTR1800017573, http://www.chictr.org.cn). The study design was described specifically elsewhere [24, 31].

**Figure 1:**
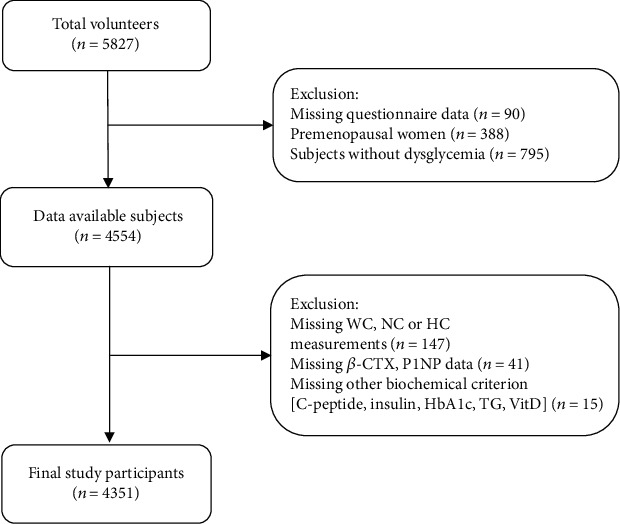
Flow chart of study participant selection (inclusion and exclusion).

After being informed about the details of the study, participants gave their written consent for involvement. Initially, 5827 volunteers (18-99 years old) who had lived in their current residence for ≥6 months were included, and those who had an acute illness and severe communication problems were excluded. Subjects missing questionnaire data (*n* = 90) or without dysglycemia (*n* = 795) were then excluded. The bone metabolism of premenopausal women (*n* = 388) was complex, and we eliminated their data to ensure that the results were accurate. A woman was considered postmenopausal if she confirmed menopause on the questionnaire or was >60 years old or >55 years old with follicle − stimulating hormone ≥ 25 IU/L. Participants who were missing waist, neck, or hip circumference measurements (*n* = 147), *β*-CTX or P1NP data (*n* = 41), or other biochemical criteria (*n* = 15) were excluded. In total, 4351 participants were involved in the final analysis (Figure 1).

### 2.2. Data Collection

The data collection was carried out by experienced staff who had been involved in the Survey on Prevalence in East China for Metabolic Diseases and Risk Factors (SPECT-China) [32]. All personnel underwent an initial certification process on the procedures and specifications of this project. Information on demographic characteristics, main complaints, previous personal and family medical history, and risk elements in daily life was accessed by a well-designed questionnaire.

### 2.3. Diagnostic Criteria

T2DM was determined using a previous diagnosis made by a healthcare professional, an FPG level ≥ 7.0 mmol/L or s, in accordance with the criteria of the American Diabetes Association. Hypertension was defined as systolic blood pressure ≥ 140 mmHg, diastolic blood pressure ≥ 90 mmHg, or a previous diagnosis of hypertension by a healthcare professional. Current smoking was defined as having smoked at least 100 cigarettes over a lifetime and still smoking at present [33].

### 2.4. Biochemical Measurements

We followed the methods of Guo et al. in 2020 [24, 31]. Venous blood was collected from the subjects between 6:00 and 10:00 a.m. after fasting since 22:00 p.m. the day before. The serum samples were aliquoted and frozen at -20°C and then sent to a central laboratory within 2-4 hours.

*β*-CTX, OC, and P1NP were detected with a chemiluminescence assay (Roche E602, Switzerland). The interassay coefficients of variation were as follows: 7.60% (*β*-CTX), 1.81% (OC), and 3.30% (P1NP). Total cholesterol (TC), triglycerides (TG), high-density lipoprotein (HDL), low-density lipoprotein (LDL), fast plasma glucose (FPG), and glycated hemoglobin (HbA1c) were measured with a Beckman Coulter AU680 (Brea, USA). Glucagon was detected with a radioimmunoassay (SN-6105, China). Serum C-peptide was assessed by an immunoassay (ARCHITECT i2000SR, Abbott Laboratories, Chicago, IL, USA). Insulin was detected by a chemiluminescence device (Abbott ARCHITECT i2000SR, Chicago, USA). Vitamin D (Vit D) was detected using a chemiluminescence assay (ADVIA Centaur XP, Siemens, Germany).

### 2.5. Anthropometric Measurements

**Figure 2:**
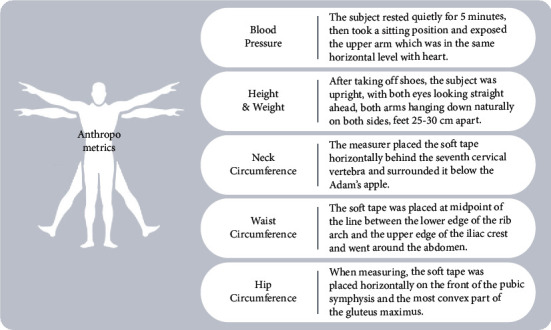
Standard anthropometric measurement methods.

When the serum samples were collected, anthropometric measurements were also obtained. Participants wore light indoor clothing. All collected parameters were accurate to 0.1 cm/0.1 kg, including blood pressure, height and weight, neck circumference (NC), WC, and hip circumference (HC) (Figure 2).

### 2.6. Obesity Indices

**Figure 3:**
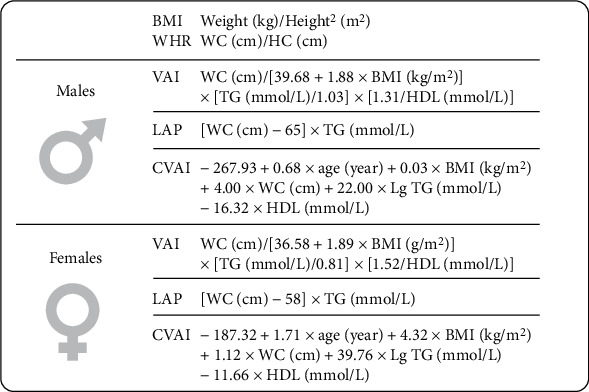
Calculation formulas of the abdominal obesity index for males and females.

BMI is widely used as an indicator of weight grouping. In accordance with the Cooperative Meta-Analysis Group of the Working Group on Obesity in China criteria, a BMI < 24 kg/m^2^ was considered normal, while a BMI ≥ 24 kg/m^2^ was defined as overweight/obesity [34]. In addition, many indicators are currently used clinically to assess the progression of abdominal obesity, such as waist-to-hip ratio (WHR), visceral adiposity index (VAI), lipid accumulation product (LAP), and Chinese visceral adiposity index (CVAI) [35–38]. Due to differences in the calculation formulas between males and females (Figure 3), the two sexes were also divided into separate groups to perform a rigorous discussion.

### 2.7. Statistical Analysis

The results were processed using IBM SPSS Statistics, Version 25 (IBM Corporation, Armonk, NY, USA). Significance was declared at a two-sided 0.05 level, unless otherwise specified. Continuous variables were expressed as the mean ± standard deviation (SD), and categorical variables were presented as percentages (%). The Mann–Whitney test and the chi-square test were used to assess the general characteristics of all male and female participants. For the association between the abdominal obesity indices and BTMs, the model was adjusted for age, TC, TG, HDL, LDL, FPG, HbA1c, glucagon, C-peptide, insulin, Vit D, current smoking, and hypertension. Multiple linear regression coefficients were determined to build a statistical model. In the analyses, the concentrations of the BTMs were naturally logarithmically transformed to follow an approximately normal distribution. To further reveal the relationship between abdominal obesity indicators concentration with BTM level, the *P* for trend was calculated by modeling the quartiles, coded as 1, 2, 3, and 4, as a continuous variable. Data are presented as *β* coefficients and 95% confidence intervals (CIs). The model was adjusted for the same correlative factors listed above.

## 3. Results

### 3.1. General Characteristics of All Male and Female Participants

Overall, 2004 males and 2347 females with diabetes were involved in the analyses. The baseline characteristics of all participants were displayed in Table 1. The average age was 67.64 ± 8.73 years for males and 67.15 ± 8.62 years for females. Between the two groups, there were significant differences in the abdominal obesity indices (NC, WC, HC, VAI, LAP, WHR, and CVAI) and the three BTMs (*β*-CTX, OC, and P1NP). Likewise, there were significant differences in the adjustment factors, such as TC, HDL, LDL, HbA1c, glucagon, Vit D, and smoking habits.

### 3.2. Association of Abdominal Obesity Indices with BTMs in T2DM Patients

The associations between the abdominal obesity indices and BTMs in T2DM populations were presented in Table 2. The model was adjusted for age, TC, TG, HDL, LDL, FPG, HbA1c, glucagon, C-peptide, insulin, Vit D, current smoking, and hypertension. In the multiple linear regression analyses, the abdominal obesity indices yielded a strongly significantly negative association of *β*-CTX and OC in the two groups, indicating a suppression effect on bone metabolism.

Among males, the results revealed five negative correlative factors with *β*-CTX (BMI, WC, LAP, WHR, and CVAI). Five indices were also negatively associated with OC (BMI, NC, WC, WHR, and CVAI). There were no significant associations between the abdominal obesity indices and P1NP. Among females, all eight indices were negatively associated with *β*-CTX. Seven indices had negative relationships with OC (BMI, NC, WC, HC, LAP, WHR, and CVAI). The VAI was also negatively correlated with P1NP.

### 3.3. *P* for Trend of Abdominal Obesity Index Concentrations with BTM Quartile Change

Table 3 further summarized the relationship between the abdominal obesity indices and the three BTMs. As the *β*-CTX and OC quartiles increased, T2DM patients were more likely to have a lower concentration of all eight indices, both in the male and female groups. As P1NP increased, only the VAI of the female group decreased.

## 4. Discussion

Through the epidemiological investigation of a large sample of people in East China, our study concluded that in diabetic populations, abdominal obesity had an obviously negative correlation with bone metabolism. The abdominal obesity indices were associated with both skeletal destruction and formation. In routine clinical practice, such easily obtained measurements can be used as a preliminary screening method and relevant factors for bone metabolic abnormalities at no additional cost but may be more valuable for postmenopausal women in T2DM patients.

Abdominal obesity is referred to a particular accumulation of adipose tissue in the abdomen, which intuitively manifested as an increased WC and was often accompanied by visceral fat sedimentation. According to the WS/T 428-2013 “Criteria of weight for adults,” a male WC ≥ 90 cm and a female WC ≥ 85 cm were defined as abdominal obesity. In 2012, the prevalence among Chinese adult residents was 25.7%, of whom male was 26.0% and female was 25.3% [39]. How fat distribution and the ensuing metabolic changes affected bone generation was not that clear [40]. It could be explained by the local inflammatory action, i.e., 11*β*-hydroxysteroid dehydrogenase type 1 (11*β*-HSD1) [41]. The enzyme's local expression and activation were upregulated by topical proinflammatory cytokines, converting osteoblasts into adipose tissue [42]. This response constituted adipocyte accumulation in the bone marrow and damaged skeletal microarchitecture [43]. Visceral adipose tissue (VAT) was metabolically more active than subcutaneous adipose tissue (SAT) [39], which might be the reason why VAT was associated with a relatively serious bone phenotype, while SAT was not significantly associated with abnormal bone metabolism.

BMI was the most frequently used index to assess overweight or obesity [44]. Although the definition was simple and numerous studies have linked it to obesity-related complications, BMI may misclassify the risks of certain individuals because it did not take into account body composition or fat distribution. Thus, many other indices have been established to estimate abdominal obesity. As the earliest and most stable evaluation index, WC has been widely used in the estimation and even diagnosis of clinical diseases, such as metabolic syndrome [45–47]. Several studies have demonstrated its correlation with metabolic abnormalities, although the relationship with bone metabolism had rarely been reported. A researcher from Denmark who enrolled 64 subjects with abdominal obesity found a negative correlation between insulin resistance, WC, and OC [48]. Another study performed in 382 Iranian postmenopausal women showed that low OC levels had significant associations with elevated blood glucose and elevated WC [49]. Likewise, in our study, WC was significantly negatively correlated with *β*-CTX and OC in both the male and female groups, but no evident correlation was found between WC and P1NP in either group. Both OC and P1NP represent bone formation, and OC might be more sensitive to obesity aggregation than P1NP. NC can reflect the extent of SAT accumulation in the upper body and has been shown to be associated with insulin resistance, cardiovascular risk factors [50, 51], and polycystic ovary syndrome [52]. Recent studies in Chinese populations have confirmed a close correlation between NC and VAT [53]. Its measurement was more facilitating than WC and was less susceptible to eating and seasonal influences. Recently, WHR has been suggested as an assessment index with a simple calculating method and has shown predictive value for overweight and obesity [54–56]. WHR could predict all-cause death [57] and was the first anthropometric measure applied in clinical community work to determine abdominal obesity [58].

The VAI, proposed in 2010, was characterized as a comprehensive aggregate of BMI, WC, TG, and HDL. The formulas were based on an adipose distribution model established by Marco's team from an Italian community [59]. VAI was considered a ponderable indicator for assessing VAT function and distribution, consistent with magnetic resonance imaging (MRI) estimations [59, 60]. In our research, VAI was the only indicator relevant to P1NP in the female group, revealing a strong connection with bone metabolism. Given the results of previous studies on VAI and considering the discrepancies caused by ethnicity, some scholars have proposed the CVAI as more suitable for the evaluation of Chinese populations [36, 37, 61]. However, at present, barely no research has investigated the association between CVAI and bone metabolism in Chinese T2DM patients, and this research served as a modest impetus to others to provide their valuable contributions.

The LAP was proposed by data from the Third National Health and Nutrition Survey in the United States [62]. Its calculations combined WC and TG, which were more scientifically sound than the basal measurements and have been shown to be closely related to dysglycemia and cardiovascular disease [63]. However, there was no unified conclusion on the critical value or tangent point of the prediction of metabolic abnormalities given differences in the ethnicities, degrees of obesity, and underlying characteristics of the study subjects [64].

Previously, body fat distribution was detected by DXA, computed tomography (CT), MR imaging (MRI), and MR spectroscopy (MRS) [65, 66]. While DXA can only distinguish bone, fat, and lean soft tissue [67], CT can differentiate adipose tissue volumes (i.e., VAT and SAT) and adipose deposition (e.g., between the liver and skeletal muscle) [65, 66]. Furthermore, MRI and MRS allowed dedicated phenotyping via the assessment of sophisticated parameters [66, 68]. But a cohort study of 1179 enrolled participants from China showed that visceral fat area measured using MRI was not associated with OC, fibroblast growth factor 23, etc. [69]. The contradictory results indicated that numerous unknowns were to be grope for. However, due to their radiation exposure, time-consuming natures, high costs, and restrictive requirements on the site and instruments, the actual utilization rate of the inspection methods remained limited. Although it was not suggested that T2DM patients be referred for central obesity anthropometric measurements to replace the conventional bone screening, the ability to identify the visceral fat content from an assessment of abdominal obesity indicators obtained during routine physical examinations could provide additional benefit in revealing osteodysfunction risk that was available at no incremental cost [22].

Limitations to this analysis include the restricted scope of the investigated population's residence. The enrolled subjects came from East China only, and the results need to be prudently generalized to other ethnic groups or regions. Second, it would be more convincing to measure BMD along with the BTMs to elucidate the trends in bone density variation. Although no causal association between abdominal obesity and bone generation can be drawn from the present cross-sectional study, future exploration of a potential “T2DM–obesity–osteodysfunction” chain of action is of immense appeal.

## 5. Conclusion

The bones of the human body were in a state of dynamic equilibrium. The present study demonstrated that in T2DM patients, abdominal obesity had an obviously negative correlation with bone metabolism. In both males and females, the abdominal obesity indices were significantly negatively associated with skeletal destruction (*β*-CTX) and formation (OC), while among females, the VAI was the only indicator relevant to P1NP. In routine clinical practice, such easily obtained measurements and indices can be calculated from concise formulas and could be used as a preliminary screening method and relevant factors for osteodysfunction risk at no incremental cost and may be of particular value for postmenopausal women in T2DM patients.

## Figures and Tables

**Table 1:** General characteristics of all male and female participants.

Characteristics	Male	Female	*P*
Subjects	2004	2347	/
Age, years	67.64 ± 8.73	67.15 ± 8.62	0.061
BMI (kg/m^2^)	24.99 ± 3.30	24.91 ± 3.84	0.430
NC (cm)	39.81 ± 3.12	36.06 ± 3.00	<0.001
WC (cm)	92.37 ± 9.01	88.54 ± 9.97	<0.001
HC (cm)	99.74 ± 6.86	97.89 ± 8.60	<0.001
VAI	2.60 ± 3.82	3.41 ± 3.83	<0.001
LAP	53.67 ± 59.11	61.12 ± 54.07	<0.001
WHR	0.93 ± 0.06	0.90 ± 0.07	<0.001
CVAI	134.40 ± 40.15	127.98 ± 34.32	<0.001
*β*-CTX (ng/mL)	0.19 ± 0.10	0.23 ± 0.11	<0.001
OC (ng/mL)	10.20 ± 4.91	13.02 ± 6.14	<0.001
P1NP (ng/mL)	38.46 ± 20.90	47.80 ± 20.10	<0.001
TC (mmol/L)	4.81 ± 1.10	5.34 ± 1.22	<0.001
TG (mmol/L)	1.87 ± 1.76	1.94 ± 1.49	0.145
HDL (mmol/L)	1.11 ± 0.25	1.29 ± 0.30	<0.001
LDL (mmol/L)	3.00 ± 0.79	3.27 ± 0.88	<0.001
FPG (mmol/L)	7.87 ± 2.40	7.75 ± 2.53	0.118
HbA1c, %	7.60 ± 1.43	7.42 ± 1.38	<0.001
Glucagon (pg/mL)	162.70 ± 82.40	178.73 ± 98.68	<0.001
C-peptide (ng/mL)	1.62 ± 0.84	1.67 ± 0.86	0.051
Insulin (pmol/L)	75.10 ± 135.24	83.11 ± 139.29	0.055
VitD (nmol/L)	42.62 ± 14.70	39.36 ± 13.67	<0.001
Smoking, %	36	2	<0.001
Hypertension, %	60	62	0.128

Continuous variables were expressed as mean ± standard deviation (SD). Categorical variables were presented as percentages (%). The Mann–Whitney test and the chi-square test were used. Abbreviations: BMI: body mass index; NC: neck circumference; WC: waist circumference; HC: hip circumference; VAI: visceral adiposity index; LAP: lipid accumulation product; WHR: waist to hip ratio; CVAI: Chinese visceral adiposity index; *β*-CTX: *β*-C-terminal cross-linking telopeptide of type I collagen; OC: osteocalcin; P1NP: procollagen type 1 N-terminal propeptide; TC: total cholesterol; TG: triglycerides; HDL: high-density lipoprotein; LDL: low-density lipoprotein; FPG: fast plasma glucose; HbA1c: glycated hemoglobin; VitD: Vitamin D.

**Table:** (a) Multiple linear regression analysis of abdominal obesity index and BTMs (male)

BTMs	Index					
	*β*-CTX	*P*	OC	*P*	P1NP	*P*
	*Β* (95% CI)		*Β* (95% CI)		*Β* (95% CI)	
BMI	-.008 (-.014, -.002)	0.006	-.009 (-.014, -.004)	<0.001	0.001 (-.004, 0.006)	0.643
NC	-.004 (-.010, 0.003)	0.258	-.007 (-.012, -.002)	0.005	0.003 (-.002, 0.009)	0.252
WC	-.004 (-.006, -.002)	<0.001	-.005 (-.007, -.003)	<0.001	0.001 (-.001, 0.003)	0.265
HC	-.001 (-.004, 0.002)	0.522	-.002 (-.004, 0.000)	0.111	0.001 (-.001, 0.004)	0.396
VAI	-.004 (-0.09, 0.002)	0.172	-.001 (-.005, 0.003)	0.686	-.002 (-.006, 0.003)	0.492
LAP	0.000 (-0.001, 0.000)	0.025	0.000 (0.000, 0.000)	0.188	0.000 (0.000, 0.000)	0.723
WHR	-1.055 (-1.430, -.681)	<0.001	-1.137 (-1.442, -.832)	<0.001	0.126 (-.204, 0.457)	0.454
CVAI	-.001 (-.001, 0.000)	0.001	-.001 (-.001, -.001)	<0.001	0.000 (0.000, 0.001)	0.183

**Table:** (b) Multiple linear regression analysis of abdominal obesity index and BTMs (female)

BTMs	Index					
	*β*-CTX	*P*	OC	*P*	P1NP	*P*
	*Β* (95% CI)		*Β* (95% CI)		*Β* (95% CI)	
BMI	-.014 (-.019, -.009)	<0.001	-.014 (-.018, -.010)	<0.001	-.001 (-.005, 0.004)	0.741
NC	-.012 (-.019, -.006)	<0.001	-.014 (-.019, -.009)	<0.001	-.001 (-.007, 0.004)	0.675
WC	-.005 (-.007, -.003)	<0.001	-.004 (-.006, -.003)	<0.001	-.001 (-.002, 0.001)	0.481
HC	-.005 (-.007, -.003)	<0.001	-.004 (-.006, -.002)	<0.001	0.000 (-.002, 0.002)	0.849
VAI	0.018 (0.000, 0.036)	0.044	0.014 (-.001, 0.029)	0.061	0.021 (0.005, 0.037)	0.010
LAP	-.002 (-.002, -.001)	<0.001	-.001 (-.002, -.001)	<0.001	0.000 (-.001, 0.001)	0.921
WHR	-.296 (-.563, -.028)	0.030	-.296 (-.516, -.075)	0.009	-.171 (-.407, 0.065)	0.156
CVAI	-.002 (-.003, -.001)	<0.001	-.002 (-.003, -.001)	<0.001	0.000 (-.001, 0.000)	0.345

The model was adjusted for age, TC, TG, HDL, LDL, FPG, HbA1c, glucagon, C-peptide, insulin, VitD, current smoking, and hypertension. The concentrations of BTMs were naturally logarithmically transformed to follow an approximately normal distribution. The abbreviations were the same with Table 1.

**(a):** 

*β*-CTX	Quartiles				*P* for trend
	1	2	3	4	
*Male*					
BMI	Ref.	-.058 (-.112, -.003)	-.045 (-.100, 0.010)	-.082 (-.137, -.026)	<0.001
WC	Ref.	-.066 (-.120, -.011)	-.027 (-.082, 0.029)	-.103 (-.161, -.046)	<0.001
NC	Ref.	-.021 (-.071, 0.028)	-.022 (-.077, 0.033)	-.032 (-.090, 0.025)	<0.001
HC	Ref.	0.012 (-.042, 0.067)	0.027 (-.026, 0.080)	-.018 (-.073, 0.036)	<0.001
VAI	Ref.	-.037 (-.092, 0.018)	0.007 (-.048, 0.062)	-.051 (-.107, 0.004)	<0.001
LAP	Ref.	0.013 (-.042, 0.068)	-.023 (-.078, 0.032)	-.053 (-.109, 0.003)	<0.001
WHR	Ref.	-.080 (-.135, -.024)	-.112 (-.169, -.054)	-.128 (-.188, -.068)	<0.001
CVAI	Ref.	-.050 (-.106, 0.005)	-.043 (-.099, 0.012)	-.078 (-.135, -.021)	<0.001
*Female*					
BMI	Ref.	-.036 (-.087, 0.016)	-.084 (-.136, -.032)	-.122 (-.177, -.068)	<0.001
WC	Ref.	-.047 (-.097, 0.003)	-.095 (-.146, -.044)	-.126 (-.179, -.073)	<0.001
NC	Ref.	0.006 (-.041, 0.053)	-.053 (-.106, 0.000)	-.084 (-.139, -.029)	<0.001
HC	Ref.	-.002 (-.052, 0.047)	-.064 (-.114, -.013)	-.089 (-.143, -.035)	<0.001
VAI	Ref.	-.012 (-.069, 0.046)	-.064 (-.129, 0.002)	-.094 (-.178, -.011)	0.013
LAP	Ref.	-.015 (-.069, 0.038)	-.108 (-.165, -.050)	-.132 (-.201, -.063)	<0.001
WHR	Ref.	-.029 (-.081, 0.022)	-.048 (-.100, 0.004)	-.057 (-.110, -.004)	0.025
CVAI	Ref.	-.053 (-.106, 0.001)	-.094 (-.153, -.036)	-.175 (-.241, -.109)	<0.001

**(b):** 

OC	Quartiles				P for trend
	1	2	3	4	
*Male*					
BMI	Ref.	-.062 (-.107, -.017)	-.045 (-.089, 0.000)	-.084 (-.129, -.038)	<0.001
WC	Ref.	-.049 (-.094, -.005)	-.042 (-.087, 0.003)	-.115 (-.162, -.068)	<0.001
NC	Ref.	-.041 (-.081, 0.000)	-.025 (-.070, 0.020)	-.068 (-.115, -.021)	<0.001
HC	Ref.	0.004 (-.041, 0.048)	0.040 (-.003, 0.083)	-.038 (-.083, 0.007)	<0.001
VAI	Ref.	-.050 (-.095, -.005)	-.026 (-.071, 0.020)	-.037 (-.082, 0.008)	<0.001
LAP	Ref.	-.034 (-.079, 0.011)	-.059 (-.104, -.014)	-.063 (-.109, -.018)	<0.001
WHR	Ref.	-.049 (-.094, -.003)	-.057 (-.104, -.010)	-.131 (-.180, -.082)	<0.001
CVAI	Ref.	-.042 (-.088, 0.003)	-.050 (-.096, -.005)	-.097 (-.144, -.051)	<0.001
*Female*					
BMI	Ref.	-.029 (-.071, 0.013)	-.069 (-.112, -.026)	-.117 (-.162, -.072)	<0.001
WC	Ref.	-.042 (-.084, -.001)	-.086 (-.128, -.044)	-.111 (-.155, -.067)	<0.001
NC	Ref.	-.009 (-.048, 0.030)	-.027 (-.070, 0.017)	-.108 (-.154, -.063)	<0.001
HC	Ref.	0.002 (-.039, 0.043)	-.050 (-.091, -.008)	-.080 (-.125, -.036)	<0.001
VAI	Ref.	-.032 (-.079, 0.016)	-.061 (-.115, -.007)	-.089 (-.158, -.020)	0.011
LAP	Ref.	-.027 (-.071, 0.017)	-.086 (-.133, -.038)	-.108 (-.165, -.051)	<0.001
WHR	Ref.	-.055 (-.097, -.012)	-.055 (-.098, -.012)	-.066 (-.109, -.022)	0.008
CVAI	Ref.	-.034 (-.078, 0.011)	-.082 (-.130, -.034)	-.147 (-.201, -.093)	<0.001

**(c):** 

P1NP	Quartiles				P for trend
	1	2	3	4	
*Male*					
BMI	Ref.	-.012 (-.061, 0.036)	0.022 (-.026, 0.070)	0.020 (-.028, 0.069)	0.181
WC	Ref.	-.031 (-.079, 0.017)	0.017 (-.031, 0.066)	0.023 (-.028, 0.073)	0.104
NC	Ref.	-.015 (-.058, 0.029)	0.026 (-.022, 0.074)	0.011 (-.039, 0.061)	0.143
HC	Ref.	0.017 (-.031, 0.065)	0.048 (0.001, 0.094)	0.012 (-.036, 0.060)	0.121
VAI	Ref.	-.011 (-.059, 0.037)	0.007 (-.041, 0.056)	0.001 (-.047, 0.050)	0.149
LAP	Ref.	0.019 (-.029, 0.067)	0.009 (-.040, 0.058)	0.008 (-.041, 0.057)	0.163
WHR	Ref.	0.011 (-.039, 0.060)	0.018 (-.032, 0.069)	0.042 (-.011, 0.094)	0.172
CVAI	Ref.	-.027 (-.076, 0.021)	0.006 (-.043, 0.054)	0.036 (-.014, 0.086)	0.163
*Female*					
BMI	Ref.	0.008 (-.037, 0.054)	-.002 (-.049, 0.044)	0.006 (-.042, 0.054)	0.947
WC	Ref.	-.005 (-.050, 0.040)	-.036 (-.081, 0.009)	-.017 (-.064, 0.031)	0.387
NC	Ref.	0.025 (-.016, 0.067)	0.023 (-.023, 0.070)	-.003 (-.052, 0.045)	0.795
HC	Ref.	0.027 (-.017, 0.071)	0.007 (-.038, 0.052)	0.022 (-.026, 0.070)	0.574
VAI	Ref.	-.022 (-.072, 0.029)	-.074 (-.131, -.016)	-.082 (-.156, -.008)	0.013
LAP	Ref.	0.011 (-.036, 0.058)	-.040 (-.091, 0.011)	-.039 (-.100, 0.023)	0.087
WHR	Ref.	-.024 (-.069, 0.022)	-.030 (-.076, 0.015)	-.027 (-.074, 0.020)	0.339
CVAI	Ref.	-.016 (-.064, 0.031)	-.022 (-.074, 0.030)	-.025 (-.083, 0.034)	0.416

Data are presented as B coefficients and 95% CI. The model was adjusted for the same correlative factors as in Table 2. The concentrations of BTMs were naturally logarithmically transformed. Ref: reference. The abbreviations were the same with Table 1.

## Data Availability

The datasets used to get the conclusion are available online. The data were retrieved from the METAL study (Environmental Pollutant Exposure and Metabolic Diseases in Shanghai) http://www.chictr.org.cn, ChiCTR1800017573.

## Abbreviations

T2DM: Diabetes mellitus type 2
BMD: Bone mineral density
WC: Waist circumference
BMI: Body mass index
BTMs: Bone turnover markers
*β*-CTX: *β*-C-terminal crosslinking telopeptide of type I collagen
OC: Osteocalcin
P1NP: Procollagen type 1 N-terminal propeptide
TC: Total cholesterol
TG: Triglycerides
HDL: High-density lipoprotein
LDL: Low-density lipoprotein
FPG: Fast plasma glucose
HbA1c: Glycated hemoglobin
Vit D: Vitamin D
NC: Neck circumference
HC: Hip circumference
WHR: Waist-to-hip ratio
VAI: Visceral adiposity index
LAP: Lipid accumulation product
CVAI: Chinese visceral adiposity index
SD: Standard deviation
CI: Confidence interval
11*β*-HSD1: 11*β*-hydroxysteroid dehydrogenase type 1
VAT: Visceral adipose tissue
SAT: Subcutaneous adipose tissue
MRI: Magnetic resonance imaging
DXA: Dual-energy X-ray absorptiometry
CT: Computed tomography
MRS: MR spectroscopy.

## References

[1] Kaur J. (2014). A comprehensive review on metabolic syndrome. *Cardiology Research & Practice*.

[2] Heilmeier U., Patsch J. (2016). Diabetes and bone. *Seminars in Musculoskeletal Radiology*.

[3] Liu Y. H., Xu Y., Wen Y. B. (2013). Association of weight-adjusted body fat and fat distribution with bone mineral density in middle-aged Chinese adults: a cross-sectional study. *PLoS One*.

[4] Ravn P., Cizza G., Bjarnason N. H. (1999). Low body mass index is an important risk factor for low bone mass and increased bone loss in early postmenopausal women. *Journal of Bone & Mineral Research*.

[5] Cao J. J. (2011). Effects of obesity on bone metabolism. *Journal of Orthopaedic Surgery and Research*.

[6] Barroso L. N., Farias D. R., Soares-Mota M. (2018). Waist circumference is an effect modifier of the association between bone mineral density and glucose metabolism. *Archives of Endocrinology and Metabolism*.

[7] Csoo R. (2019). Guidelines for the diagnosis and management of primary osteoporosis (2017). *Journal of Osteoporosis*.

[8] Ines B. (2017). Bone mineral density in relation to metabolic syndrome components in postmenopausal women with diabetes mellitus type 2. *Acta Clinica Croatica*.

[9] Ng A. C., Melton L. J., Atkinson E. J. (2013). Relationship of adiposity to bone volumetric density and microstructure in men and women across the adult lifespan. *Bone*.

[10] Cohen A., Dempster D. W., Recker R. R. (2013). Abdominal fat is associated with lower bone formation and inferior bone quality in healthy premenopausal women: a transiliac bone biopsy study. *The Journal of Clinical Endocrinology and Metabolism*.

[11] Chung W., Lee J., Ryu O. H. (2014). Is the negative relationship between obesity and bone mineral content greater for older women?. *Journal of Bone & Mineral Metabolism*.

[12] Egger G., Dixon J. (2014). Beyond obesity and lifestyle: a review of 21st century chronic disease determinants. *BioMed Research International*.

[13] Kling J. M., Clarke B. L., Sandhu N. P. (2014). Osteoporosis prevention, screening, and treatment: a review. *Journal of Women’s Health*.

[14] Yoo H. J., Park M. S., Yang S. J. (2012). The differential relationship between fat mass and bone mineral density by gender and menopausal status. *Journal of Bone & Mineral Metabolism*.

[15] Duclos M. (2016). Osteoarthritis, obesity and type 2 diabetes: the weight of waist circumference. *Annals of Physical and Rehabilitation Medicine*.

[16] Kindler J. M., Kelly A., Khoury P. R., Levitt Katz L. E., Urbina E. M., Zemel B. S. (2020). Bone mass and density in youth with type 2 diabetes. *Obesity, and Healthy Weight*.

[17] Jia X., Liu L., Wang R. (2020). Relationship of two-hour plasma glucose and abdominal visceral fat with bone mineral density and bone mineral content in women with different glucose metabolism status. *Diabetes, Metabolic Syndrome and Obesity: Targets and Therapy*.

[18] Cerhan J. R., Moore S. C., Jacobs E. J. (2014). A pooled analysis of waist circumference and mortality in 650,000 adults. *Mayo Clinic Proceedings*.

[19] Batsis J. A., Sahakyan K. R., Rodriguez-Escudero J. P., Bartels S. J., Lopez-Jimenez F. (2014). Normal weight obesity and functional outcomes in older adults. *European Journal of Internal Medicine*.

[20] Batsis J. A., Zbehlik A. J., Scherer E. A., Barre L. K., Bartels S. J. (2015). Normal weight with central obesity, physical activity, and functional decline: data from the osteoarthritis initiative. *Journal of the American Geriatrics Society*.

[21] Gu L. J., Lai X. Y., Wang Y. P., Zhang J. M., Liu J. P. (2019). A community-based study of the relationship between calcaneal bone mineral density and systemic parameters of blood glucose and lipids. *Medicine*.

[22] Leslie W. D., Ludwig S. M., Suzanne M. (2009). Abdominal fat from spine dual-energy x-ray absorptiometry and risk for subsequent diabetes. *Journal of Clinical Endocrinology & Metabolism*.

[23] Qu Y., Kang M. Y., Dong R. P., Zhao J. W. (2016). Correlations between abnormal glucose metabolism and bone mineral density or bone metabolism. *Medical Science Monitor International Medical Journal of Experimental & Clinical Research*.

[24] Guo H., Wang C., Jiang B. (2021). Association of insulin resistance and *β*-cell function with bone turnover biomarkers in dysglycemia patients. *Frontiers in Endocrinology*.

[25] Daniele M., Biggs M. L., Walker M. D. (2018). Biochemical markers of bone turnover and risk of incident diabetes in older women: the cardiovascular health study. *Diabetes Care*.

[26] van Bommel E. J., de Jongh R. T., Brands M. (2018). The osteoblast: linking glucocorticoid-induced osteoporosis and hyperglycaemia? A post-hoc analysis of a randomised clinical trial. *Bone*.

[27] Brown J. P., Albert C., Nassar B. A. (2009). Bone turnover markers in the management of postmenopausal osteoporosis. *Clinical Biochemistry*.

[28] Eastell R., Szulc P. (2017). Use of bone turnover markers in postmenopausal osteoporosis. *Lancet Diabetes & Endocrinology*.

[29] Wan H., Zhang K., Wang Y. (2020). The associations between gonadal hormones and serum uric acid levels in men and postmenopausal women with diabetes. *Frontiers in Endocrinology*.

[30] Wan H., Wang Y., Chen Y. (2020). Different associations between serum urate and diabetic complications in men and postmenopausal women. *Diabetes Research and Clinical Practice*.

[31] Guo H., Sui C., Ge S. (2022). Positive association of glucagon with bone turnover markers in type 2 diabetes: a cross-sectional study. *Diabetes/Metabolism Research and Reviews*.

[32] Wang N., Wang X., Li Q. (2017). The famine exposure in early life and metabolic syndrome in adulthood. *Clinical Nutrition*.

[33] Bi Y., Xu Y., Li M. (2013). Prevalence and control of diabetes in Chinese adults: the China metabolic risk factor study. *Circulation*.

[34] Zhou B.-F. (2002). Effect of body mass index on all-cause mortality and incidence of cardiovascular diseases--report for meta-analysis of prospective studies open optimal cut-off points of body mass index in Chinese adults. *Biomedical & Environmental Sciences*.

[35] Zhao L., Huang G., Xia F. (2018). Neck circumference as an independent indicator of visceral obesity in a Chinese population. *Lipids in Health and Disease*.

[36] Xia M. F., Lin H. D., Chen L. Y. (2018). Association of visceral adiposity and its longitudinal increase with the risk of diabetes in Chinese adults: a prospective cohort study. *Diabetes/metabolism Research & Reviews*.

[37] Wu J., Gong L., Li Q. (2017). A novel visceral adiposity index for prediction of type 2 diabetes and pre-diabetes in Chinese adults: a 5-year prospective study. *Scientific Reports*.

[38] Yang G. R., Yuan S. Y., Fu H. J. (2010). Neck circumference positively related with central obesity, overweight, and metabolic syndrome in Chinese subjects with type 2 diabetes: Beijing community diabetes study 4. *Diabetes Care*.

[39] Zhao Y., Hu Y., Smith J. P., Strauss J., Yang G. (2014). Cohort profile: the China Health and Retirement Longitudinal Study (CHARLS). *International Journal of Epidemiology*.

[40] Premaor M. O., Compston J. E., Fina Avilés F. (2013). The association between fracture site and obesity in men: a population-based cohort study. *Journal of Bone and Mineral Research: the Official Journal of the American Society for Bone and Mineral Research*.

[41] Savvidis C., Tournis S., Dede A. D. (2018). Obesity and bone metabolism. *Hormones*.

[42] Cooper M. S., Walker E. A., Bland R., Fraser W. D., Hewison M., Stewart P. M. (2000). Expression and functional consequences of 11*β*-hydroxysteroid dehydrogenase activity in human bone. *Bone*.

[43] Cooper M. S., Bujalska I., Rabbitt E. (2001). Modulation of 11beta-hydroxysteroid dehydrogenase isozymes by proinflammatory cytokines in osteoblasts: an autocrine switch from glucocorticoid inactivation to activation. *Journal of Bone and Mineral Research*.

[44] Pischon T., Boeing H., Hoffmann K. (2009). General and abdominal adiposity and risk of death in Europe. *Journal of Vascular Surgery*.

[45] Papaetis G. S., Papakyriakou P., Panagiotou T. N. (2015). Central obesity, type 2 diabetes and insulin: exploring a pathway full of thorns. *Archives of Medical Science Ams*.

[46] Mclaughlin T., Lamendola C., Liu A., Abbasi F. (2011). Preferential fat deposition in subcutaneous versus visceral depots is associated with insulin sensitivity. *Journal of Clinical Endocrinology & Metabolism*.

[47] Ibrahim M. M. (2010). Subcutaneous and visceral adipose tissue: structural and functional differences. *Obesity Reviews*.

[48] Gregersen S., Rakvaag E., Vestergaard P. (2020). Consumption of nutrients and insulin resistance suppress markers of bone turnover in subjects with abdominal obesity. *Bone*.

[49] Movahed A., Larijani B., Nabipour I. (2012). Reduced serum osteocalcin concentrations are associated with type 2 diabetes mellitus and the metabolic syndrome components in postmenopausal women: the crosstalk between bone and energy metabolism. *Journal of Bone & Mineral Metabolism*.

[50] Laakso M., Matilainen V., Keinänen-Kiukaanniemi S. (2002). Association of neck circumference with insulin resistance-related factors. *International Journal of Obesity*.

[51] Onat A., Hergenç G., Yüksel H. (2009). Neck circumference as a measure of central obesity: associations with metabolic syndrome and obstructive sleep apnea syndrome beyond waist circumference. *Clinical Nutrition*.

[52] Dixon J. B., O'brien P. E. (2002). Neck circumference a good predictor of raised insulin and free androgen index in obese premenopausal women: changes with weight loss. *Clinical Endocrinology*.

[53] Li H. X., Zhang F., Zhao D. (2014). Neck circumference as a measure of neck fat and abdominal visceral fat in Chinese adults. *BMC Public Health*.

[54] Ashwell M., Gunn P., Gibson S. (2012). Waist-to-height ratio is a better screening tool than waist circumference and BMI for adult cardiometabolic risk factors: systematic review and meta- analysis. *Obesity Reviews*.

[55] Shao J., Yu L., Shen X., Li D., Wang K. (2010). Waist-to-height ratio, an optimal predictor for obesity and metabolic syndrome in Chinese adults. *Journal of Nutrition Health & Aging*.

[56] Browning L. M., Hsieh S. D., Ashwell M. (2010). A systematic review of waist-to-height ratio as a screening tool for the prediction of cardiovascular disease and diabetes: 0·5 could be a suitable global boundary value. *Nutrition Research Reviews*.

[57] Srikanthan P., Seeman T. E., Karlamangla A. S. (2009). Waist-hip-ratio as a predictor of all-cause mortality in high-functioning older adults. *Annals of Epidemiology*.

[58] Rankinen T., Kim S. Y., Pérusse L., Després J. P., Bouchard C. (1999). The prediction of abdominal visceral fat level from body composition and anthropometry: ROC analysis. *International Journal of Obesity*.

[59] Amato M. C., Giordano C., Galia M. (2010). Visceral adiposity index: a reliable indicator of visceral fat function associated with cardiometabolic risk. *Diabetes Care*.

[60] Oh J., Sung Y., Lee H. J. (2013). The visceral adiposity index as a predictor of insulin resistance in young women with polycystic ovary syndrome. *Obesity*.

[61] Xia M. F., Chen Y., Lin H. D. (2016). A indicator of visceral adipose dysfunction to evaluate metabolic health in adult Chinese. *Scientific Reports*.

[62] Kahn H. S. (2005). The "lipid accumulation product" performs better than the body mass index for recognizing cardiovascular risk: a population-based comparison. *BMC Cardiovascular Disorders*.

[63] Kahn S. H. (2006). The lipid accumulation product is better than BMI for identifying diabetes: a population-based comparison. *Diabetes Care*.

[64] Ahn N., Baumeister S. E., Amann U. (2019). Visceral adiposity index (VAI), lipid accumulation product (LAP), and product of triglycerides and glucose (TyG) to discriminate prediabetes and diabetes. *Scientific Reports*.

[65] Seabolt L. A., Welch E. B., Silver H. J. (2015). Imaging methods for analyzing body composition in human obesity and cardiometabolic disease. *Annals of the New York Academy of Sciences*.

[66] Wang H., Chen Y. E., Eitzman D. T. (2014). Imaging body fat: techniques and cardiometabolic implications. *Arteriosclerosis Thrombosis & Vascular Biology*.

[67] Baum T., Cordes C., Dieckmeyer M. (2016). MR-based assessment of body fat distribution and characteristics. *European Journal of Radiology*.

[68] Thomas E. L., Parkinson J. R., Frost G. S. (2012). The missing risk: MRI and MRS phenotyping of abdominal adiposity and ectopic fat. *Obesity*.

[69] Xu Y., Ma X., Pan X., He X., Xiao Y., Bao Y. (2018). Correlations between serum concentration of three bone-derived factors and obesity and visceral fat accumulation in a cohort of middle aged men and women. *Cardiovascular Diabetology*.

